# Antioxidant and Anti-Melanogenic Activities of Heat-Treated Licorice (Wongam, *Glycyrrhiza glabra* × *G. uralensis*) Extract

**DOI:** 10.3390/cimb43020083

**Published:** 2021-09-18

**Authors:** Min Hye Kang, Gwi Yeong Jang, Yun-Jeong Ji, Jeong Hoon Lee, Su Ji Choi, Tae Kyung Hyun, Hyung Don Kim

**Affiliations:** 1 Department of Herbal Crop Research, National Institute of Horticultural and Herbal Science (NIHHS), Rural Development Administration (RDA), Eumsung 27709, Korea; mohak2@korea.kr (M.H.K.); janggy@korea.kr (G.Y.J.); jyj2842@korea.kr (Y.-J.J.); artemisia@korea.kr (J.H.L.); suji8937@korea.kr (S.J.C.); 2 Department of Industrial Plant Science and Technology, College of Agricultural, Life and Environmental Sciences, Chungbuk National University, Cheongju 28644, Korea

**Keywords:** anti-melanogenic activity, heat treatment, licorice, murine melanoma cells (B16F10)

## Abstract

Melanin is a brown or black pigment that protects skin from ultraviolet radiation and reactive oxygen species (ROS). However, overproduction of melanin is associated with lentigines, melasma, freckles and skin cancer. Licorice has shown antioxidant, anti-tumor, anti-platelet, anti-inflammatory and immunomodulatory activities and is used as a natural treatment for skin whitening. We aimed to confirm the potential of Wongam, a new cultivar of licorice developed by the Rural Development Administration (RDA), as a whitening agent in cosmetics. In addition, we verified the effect of heat treatment on the bioactivity of licorice by comparing antioxidant and anti-melanogenic activities of licorice extract before and after heating (130 °C). The heat-treated licorice extract (WH-130) showed higher radical-scavenging activities in the ABTS^+^ (2,2′-azino-bis-(3-ethylbenzothiazolin-6-sulfonic acid) diammonium salt) and DPPH (2,2-diphenyl-1-picrylhydrazyl) assays. In addition, WH-130 inhibited melanogenesis more effectively due to downregulation of tyrosinase in B16F10 melanoma cells than non-heated licorice extract. Moreover, heat treatment increased total phenolic content. In particular, isoliquiritigenin, an antioxidant and anti-melanogenic compound of licorice, was produced by heat treatment. In conclusion, WH-130, with increased levels of bioactive phenolics such as isoliquiritigenin, has potential for development into a novel skin whitening material with applications in cosmetics.

## 1. Introduction

Licorice (*Glycyrrhiza* species), a perennial herb with medicinal uses in the family Leguminosae, is widely distributed across Central Asia, China, Russia, Manchuria, Mongolia and Europe [1]. It has long been utilized as a flavoring and sweetening agent. The dried root and stolon of licorice have been used for treatment of cold, cough, asthma, fatigue and respiratory tract infections due to the biological activities of licorice including antioxidant, anti-microbial, anti-tumor, anti-platelet, anti-inflammatory and immunomodulatory activities [2,3]. The genus *Glycyrrhiza* consists of about 22 licorice species, including *G. uralensis* Fisch, *G. glabra* L. and *G. inflata* Batal.

Wongam, a new interspecific hybrid cultivar of licorice, was developed by the Rural Development Administration as a hybrid of *Glycyrrhiza glabra* x *G. uralensis* (*G. korshinski* Grig). Wongam has higher yields and better disease resistance than *G. uralensis*. Numerous studies have been conducted on the biological activities of *G. uralensis*, whereas existing research on Wongam, a new cultivar, is limited to its anti-allergic, immunomodulatory and anti-ulcer activities [1,2]. Thus, it is necessary to investigate the broad bioactivity of Wongam.

The major bioactive components in licorice root are flavonoids and triterpene saponins, including liquiritin, liquiritigenin, isoliquiritigenin (ISL) and glycyrrhizin (or glycyrrhizic acid) [4,5]. ISL is a flavonoid found in licorice that has shown various pharmaceutical activities, including anti-platelet, anti-allergic, anti-tumor, anti-inflammatory and antioxidant activities [6,7,8]. ISL is a hydrolysis product from isoliquiritin in licorice root. It can inhibit melanogenesis through the degradation of microphthalmia-associated transcription factor (MITF) by activation of the extracellular signal-regulated protein kinase (ERK) signaling pathway. Consequently, MITF degradation suppressed the expression of tyrosinase *(TYR)* and tyrosinase-related proteins *(TRP-1* and *TRP-2)* genes in SK-MEL-2 cells [9,10]. ISL can inhibit the mono- and diphenolase activities of mushroom TYR as well as the melanogenesis in melanocytes [11].

Melanin, a major pigment imparting skin color, is synthesized in epidermal melanocytes and stored in intracellular organelles called melanosomes [12]. Melanin pigments consist of two types: brown or black eumelanin and red or yellow pheomelanin [12]. Melanogenesis can be induced by external stimuli, including ultraviolet (UV) radiation, reactive oxygen species (ROS) and chemicals such as α-melanocyte-stimulating hormone (α-MSH) and isobutylmethylxanthine (IBMX), which elevates cyclic adenosine monophosphate (cAMP) levels. Melanin protects the skin against these stimuli, while its overproduction causes skin hyperpigmentation, which can lead to diseases such as skin cancer [13,14,15,16,17]. Skin-whitening agents such as kojic acid, hydroquinone, arbutin and ascorbic acid, which are TYR inhibitors, have been utilized to treat hyperpigmentation in several cosmetic products [13,18]. Melanogenesis is regulated through a variety of signaling pathways, and these signals are linked to upregulation of MITF. Activation of MITF promotes the expression of TYR and TRPs (TRP-1, TRP-2). Then, TYR catalyzes the oxidation of L-tyrosine to 3,4-dihydroxy-L-phenylalanine (L-DOPA), which is subsequently oxidized into DOPA quinone. TRP-2 converts dopachrome to 5,6-dihydroxyindole-2-carboxylic acid (DHICA) or 5,6-dihydroxyindole (DHI). Finally, DHICA and DHI are oxidized by TRP-1, resulting in melanogenesis [19].

Thermal processes affect the physicochemical and biological properties of plant extracts. They destroy cell walls and cleave covalent bonds, resulting in the release of bioactive compounds [20,21]. Heat treatment of brewers spent grain extracts above 130 °C increases total phenolic content (TPC) and total flavonoid content (TFC) levels, with corresponding increases in antioxidant activities [16,20,21]. Heating of citrus peels can lead to release of phenolic compounds and increased antioxidant activity [17].

Our main aim is to confirm the potential of Wongam as a natural skin whitening material in cosmetics. In addition, this study is also intended to prove whether heat treatment can improve the antioxidant and anti-melanogenic activities of licorice. We investigated antioxidant activities, together with TPC of Wongam extracts. Anti-melanogenic effects of Wongam extracts were verified by TYR inhibition activity and melanin content in melanoma cells.

## 2. Materials and Methods

### 2.1. Sample Preparation

Samples were prepared according to a previously reported method [22]. Licorice (Wongam, *Glycyrrhiza glabra* x *G. uralensis,* identified by Yun Ji Lee from NIHHS, RDA and registered in the Korea Medicinal Resources Herbarium with voucher number MPS006326) was cultivated and harvested at the Department of Herbal Crop Research (Eumsung, Chungcheongbuk-do, Korea) in 2018, and dried at 60 °C for 20 h. 10 kg licorice was harvested and 1 kg was ground. After mixing them, 3 samples of WC or WH were obtained, respectively. Ground licorice (5 g) was placed in a glass container with 1 mL of distilled water and treated in an autoclave (Jisico, Seoul, Korea) for 1 h at 130 °C (WH-130). Untreated control licorice (WC, 5 g) and WH-130 were separately extracted using 70% ethanol (sample:solvent, 1:50, *v:v*) for 24 h at room temperature (RT). After filtration, the extracts were evaporated under vacuum, freeze-dried and stored at −80 °C. All extracts were solubilized in dimethyl sulfoxide (DMSO) (Sigma, St. Louis, MO, USA) and methanol (Sigma, St. Louis, MO, USA).

### 2.2. Determination of Browning

The browning index of licorice extracts (WC and WH-130) was recorded based on their absorbance value at 420 nm (A_420_) measured using a microplate reader (BioTek, Winooski, VT, USA). Higher values of absorbance at 420 nm correspond to higher browning index [23,24].

### 2.3. Total Phenolic Content (TPC)

According to a previously reported method [25], TPC was measured based on the principle that Folin–Ciocalteu’s reagent is reduced to a blue chromophore comprised of a phosphotungstic-phosphomolybdenum complex depending on alkaline conditions and on the concentration of phenolic compounds. Each sample (10 μL) was mixed with 2% sodium carbonate (200 μL) and then Folin–Ciocalteu’s reagent (10 μL). The mixture was activated for 30 min at RT. Then, the absorbance was measured at 750 nm using a microplate reader (BioTek, Winooski, VT, USA). TPC was calculated from the standard curve of gallic acid and the results were expressed as gallic acid equivalent values, mg GAE g^−1^ extract.

### 2.4. Determination of Antioxidant Capacity

Radical-scavenging activity was measured using 2,2′-azino-bis-(3-ethylbenzothiazolin-6-sulfonic acid) diammonium salt (ABTS) according to a previously described method with slight modification [19,26]. ABTS radical cation solution was produced by mixing 2.6 mM potassium persulfate and 7.4 mM ABTS and allowing them to react for 24 h at RT. The ABTS solution was then diluted with distilled water to adjust absorbance to the range of 1.000–1.500. Next, 20 μL of sample was reacted with 180 μL of ABTS^+^ solution, protected from light, and the mixture of sample and solution was incubated for 30 min at RT. Absorbance was measured using a microplate reader (BioTek, Winooski, VT, USA) at 735 nm. From a trolox standard curve, the trolox equivalent antioxidant capacity (TEAC) was calculated and expressed in mg of trolox equivalent (TE) per g of dry sample.

2,2-Diphenyl-1-picrylhydrazyl (DPPH) radical solution was prepared by dissolving 0.2 mM DPPH into 99% ethanol. DPPH solution was diluted with distilled water to an absorbance at 520 nm of 1000–1500. Then, 50 μL of sample was mixed with 200 μL of DPPH solution and reacted for 30 min at RT. After reaction, absorbance of the mixture was determined at 520 nm. From the trolox standard curve, TEAC was calculated and expressed in mg TE per g of dry sample.

### 2.5. High-Performance Liquid Chromatography (HPLC) Analysis

A modified HPLC method was applied [22]. The ISL content of licorice extracts was analyzed by HPLC with a UV–visible detector (HPLC: 1200 series, Agilent Technologies, Santa Clara, CA, USA; column: SynergiTM, 250 × 4.6 mm, 4 μm, Fusion-RP 80 Å, Phenomenex, Torrance, CA, USA). The mobile phase consisted of solvent A (0.1% formic acid in water) and solvent B (0.1% formic acid in acetonitrile). The gradient program for the mobile phase was operated under the following gradient: 0–8 min (20–20% B), 8–30 min (20–38% B), 30–42 min (38–50% B), 42–47 min (50–90% B), 47–53 min (90–90% B), 53–54 min (90–20% B) and 54–60 min (20–20% B). The flow rate, detection wavelength and injection volume were set to 1.0 mL/min, 360 nm and 10 μL, respectively. The ISL standard solutions were analyzed with four different concentrations (0.05, 0.1, 0.2, 0.4 mg/mL). The calibration curve (y = ax + b) was used to determine the content of ISL. In the regression equation, x refers to the concentration of the ISL (mg mL^−1^) and y means the peak area.

### 2.6. Mushroom Tyrosinase Inhibitory Activity Assay

The TYR inhibitory activity assay was conducted using a TYR inhibitor screening kit (BioVision, Milpitas, CA, USA). Each extract (WC and WH-130) was dissolved in DMSO to a final concentration of 250 μg/mL. Kojic acid, used as a positive control, was also diluted to 250 μg/mL in DMSO. In brief, 20 μL of extract or kojic acid was combined with 50 μL of TYR enzyme solution. After incubation at RT for 10 min, 30 μL of TYR substrate solution was added to each well. The final concentration of the extracts and kojic acid was 50 μg/mL. Optical density of the wells was then measured at 510 nm in kinetic mode for 1 h with a microplate reader (BioTek, Winooski, VT, USA).

### 2.7. Cell Culture

Murine melanoma B16F10 cells were obtained from the American Type Culture Collection (ATCC, Manassas, VA, USA). B16F10 cells were cultured in Dulbecco’s modified Eagle’s medium (DMEM) supplemented with 10% fetal bovine serum (FBS), 100 units/mL of penicillin and 100 μg/mL of streptomycin (Gibco, Grand Island, NY, USA) in a humidified atmosphere containing 5% CO_2_ at 37 °C. 

### 2.8. Cell Viability Assay

The cytotoxicity of licorice extracts (WC and WH-130) to B16F10 cells was determined using the MTT [3-(4,5-dimethylthiazol-2-yl)-2,5-diphenyltetrazolium bromide] assay. B16F10 cells were cultured at 4 × 10^3^ cells/well in a 96-well plate for 24 h. Thereafter, extracts were added to the cells at concentrations of 50, 100 and 200 μg/mL and incubated at 37 °C for 48 h. Then, MTT solution was added to each well (500 μg/mL) and the plate was incubated for 1 h at 37 °C. The medium in each well was discarded and 100 μL of DMSO was added. After shaking for 30 min, the absorbance was measured at 540 nm with a microplate reader. All tests were performed in triplicate.

### 2.9. Measurement of Cellular Melanin Content

Intracellular melanin content was measured using a slightly modified version of a method previously described [27]. B16F10 melanoma cells were seeded in 6-well plates at a density of 8 × 10^4^ cells/well and incubated for 24 h. The cells were exposed to the extracts (WC and WH-130, 100 μg/mL), kojic acid (100 μg/mL) or ISL (10 μM, MilliporeSigma, Burlington, MA, United States) for 48 h in the presence of 100 μM 3-isobutyl-1-methylxanthine (IBMX). After treatment, the cells were washed with Dulbecco’s phosphate-buffered saline (DPBS) and dissolved in 200 μL of 1 N NaOH for 2 h at 90 °C. The absorbance at 405 nm was measured using a microplate reader. 

### 2.10. Cellular Tyrosinase Activity Assay

Intracellular TYR activity was measured using a slightly modified version of a previously described method [27]. B16F10 melanoma cells (8 × 10^4^ cells/well) were treated with the extracts (100 μg/mL) or kojic acid (100 μg/mL) in the presence of 100 μM IBMX for 48 h, collected and washed with DPBS. The protein samples were lysed in 1% Trion X-100 (MilliporeSigma, Burlington, MA, USA) with sodium phosphate buffer (pH 6.8) and then centrifuged at 15,000 rpm for 10 min. The supernatant fraction was collected and the protein concentration was determined using the bicinchoninic acid (BCA) assay kit (Waltham, MA, USA). A reaction mixture consisting of 30 μg of protein (adjusted to 100 μL with 1% Trion X-100) and 100 μL of 10 mM L-DOPA was added to each well of a 96-well plate. After incubation at 37 °C for 1 h, dopachrome was monitored by measuring the absorbance at 490 nm using a microplate reader. TYR activity was calculated with the following formula:

Tyrosinase activity (%) = (OD_405_ of sample/OD_405_ of control) × 100

### 2.11. Real-Time Polymerase Chain Reaction (RT-PCR)

The mRNA expression levels of melanogenesis-related genes including MITF, TYR, TRP-1 and TRP-2 were determined using a modified RT-PCR assay [28]. Briefly, B16F10 cells were seeded in 6-well plates at a density of 8 × 10^4^ cells per well and cultured for 24 h. Then, the cells were treated with extracts (100 μg/mL), kojic acid (100 μg/mL) or ISL (10 μM) for 48 h. Total RNA was isolated from the cells using TRIzol reagent (Ambion, Austin, TX, USA) and then the concentration of RNA was quantified using a microplate reader. After preparation of cDNA (2 μg) using the Reverse Transcriptase Premix Kit (Elpis Biotech, Daejeon, Korea) according to the manufacturer’s protocols, real-time PCR was performed using MITF, TYR, TRP-1, TRP-2 and β-actin primers (Table 1, Bioneer, Daejeon, Korea) with a SYBR Green kit (ProGEN, Heidelberg, Germany) in a CFX96 Real Time PCR instrument (Bio-Rad, Hercules, CA, USA). All data were normalized to levels of β-actin mRNA as a reference housekeeping gene.

### 2.12. Preparation of Cell Lysates and Western Blot Analysis

B16F10 cells were seeded in 6-well plates at a density of 8 × 10^4^ cells per well, incubated for 24 h and then exposed to extracts (100 μg/mL), kojic acid (100 μg/mL) or ISL (10 μM) for 48 h. After washing with DPBS, cells were harvested and lysed in Triton X-100 buffer (MilliporeSigma, Burlington, MA, USA) containing 1% protease and phosphatase inhibitor cocktail. The protein concentrations of whole-cell lysates were measured using a BCA assay kit (Waltham, MA, USA). Equal amounts of protein (20 μg) were loaded onto 10% sodium dodecyl sulfate polyacrylamide gels, which were run for 4 h at 70 V. Then, the proteins were transferred to a polyvinylidene fluoride (PVDF) membrane. The membrane was washed three times with Tris-buffered saline [50 mM Tris-HCl (pH 7.5), 150 mM NaCl] containing 0.1% Tween 20 (TBST) and blocked with 2% bovine serum albumin (GenDEPOT) for 1 h. Next, the membranes were incubated overnight at 4 °C with the primary antibodies (dilution 1:1000) for 4 h at room temperature. MITF, TYR, TRP-1, TPR-2 and β-actin were detected with rabbit polyclonal anti-MITF antibody (Abcam, Cambridge, UK); goat polyclonal anti-TYR antibody (Santa Cruz, Dallas, TX, USA); and mouse monoclonal anti-TRP-1, anti-TRP-2 and anti-β-actin antibodies (Santa Cruz, Dallas, TX, USA), respectively. Afterward, the membranes were washed several times with TBST and incubated with secondary antibodies (dilution 1:2000) for 1 h, namely rabbit anti-mouse IgG, mouse anti-goat IgG and goat anti-mouse IgG (Santa Cruz, Dallas, TX, USA), followed by several washes with TBST. Target proteins were detected using an enhanced chemiluminescence (ECL) reagent (Bio-Rad, Hercules, CA, USA) with the ChemiDoc Imaging System (Bio-Rad, Hercules, CA, USA). Protein band quantification was conducted using ImageJ software (version 1.52a for Windows; NIH, Rockville, MD, USA) [28].

### 2.13. Statistical Analysis

The data were analyzed using Microsoft Excel 2016 and all experimental results are presented as the mean ± standard deviation (SD) of three independent measurements. Student’s two-sample t-test was used to assess differences between the control and samples. One-way analysis of variance (ANOVA) and the Duncan test were performed using R software (Version 3.6.3) to determine the significance of each comparison at levels of *p* < 0.05 (*), *p* < 0.01 (**) and *p* < 0.001 (***). Correlation analysis was performed using Prism 5.02 (GraphPad Software, San Diego, CA, USA).

## 3. Results

### 3.1. Browning Index of Licorice Extracts

**Figure 1:**
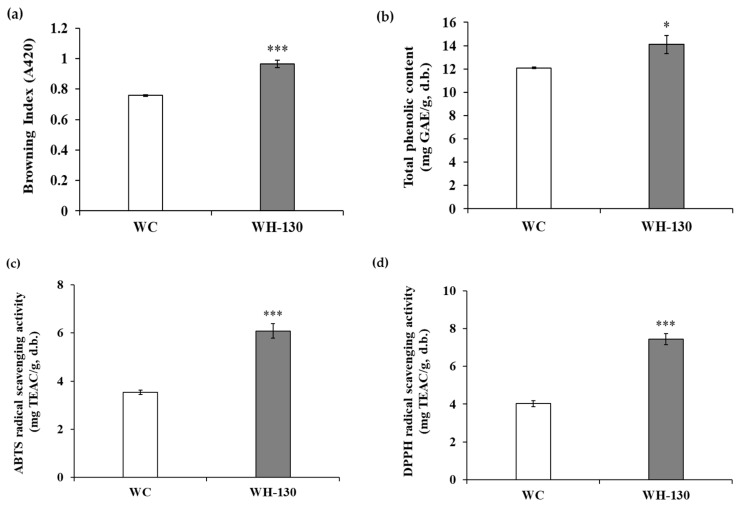
Browning index, TPC and antioxidant activities based on ABTS^+^ and DPPH assays of licorice extracts: (**a**) browning index, (**b**) TPC, (**c**) ABTS radical scavenging activity, (**d**) DPPH radical scavenging activity. TPC of tested samples are expressed as gallic acid equivalent on a dry mass basis. Radical scavenging activities of tested samples are expressed as Trolox equivalent on a dry mass basis. Values are expressed as mean ± SD from three experimental replicates. * *p* < 0.05, *** *p* < 0.001.

The degree of browning of licorice extracts is shown in Figure 1a. These results confirmed that heat-treated extract (WH-130, 0.965 ± 0.025 A.U. [absorbance units]) had much higher browning index values than non-heated extract (WC, 0.758 ± 0.005 A.U.). We expect that thermal processing increases melanoidin formation in WH-130, and that this change may be linked to various biological activities. 

### 3.2. Total Phenolic Content (TPC) of Licorice Extracts

Figure 1b shows the TPC of licorice extracts. We found a difference in TPC between WC and WH-130, as WC had a TPC of 12.093 ± 0.061 mg GAE/g extract, while WH-130 had a TPC of 14.098 ± 0.781 mg GAE/g extract. Heat treatment increased TPC of licorice extracts.

### 3.3. Radical-Scavenging Activity of Licorice Extracts

The antioxidant activity of licorice extracts is presented in Figure 1c,d. The ABTS^+^ radical-scavenging capacity was higher for WH-130 extract (6.086 ± 0.304 mg TEAC/g extract) than WC extract (3.542 ± 0.093 mg TEAC/g extract). In the DPPH assay, a similar tendency was observed. WH-130 (7.442 ± 0.292 mg TEAC/g extract) extract showed greater DPPH radical-scavenging activity than WC extract (4.033 ± 0.160 mg TEAC/g extract). These results indicate that heating can increase the antioxidant activity of extracts.

### 3.4. Isoliquiritigenin Content of Licorice Extracts

**Figure 2:**
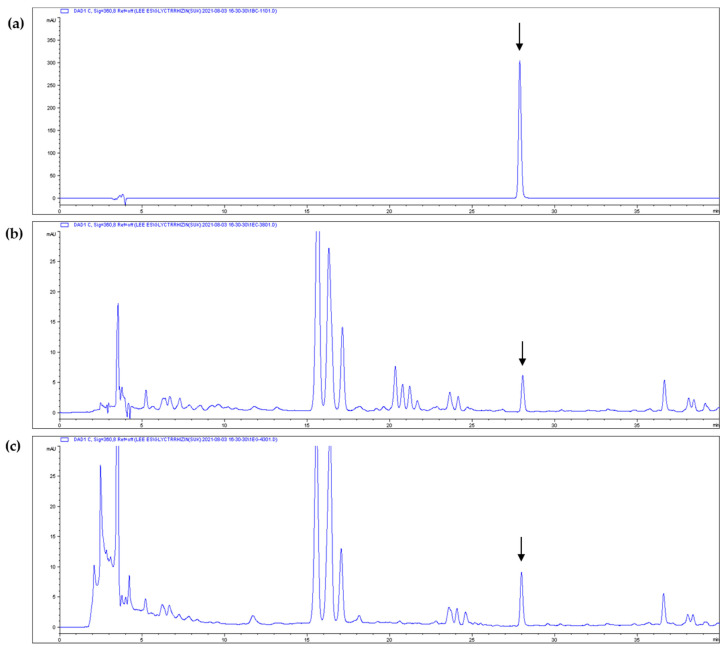
High-performance liquid chromatography (HPLC) chromatograms of isoliquiritigenin and licorice extract: (**a**) isoliquiritigenin standard, (**b**) non-heated licorice (WC) and (**c**) heat-treated licorice (WH-130). Arrows indicate the isoliquiritigenin peak.

The results showed that the ISL content of WC increased markedly after heat treatment (Table 2, Figure 2), indicating that thermal processing can affect the content of this antioxidant and anti-melanogenic compound.

### 3.5. Cell Viability

**Figure 3:**
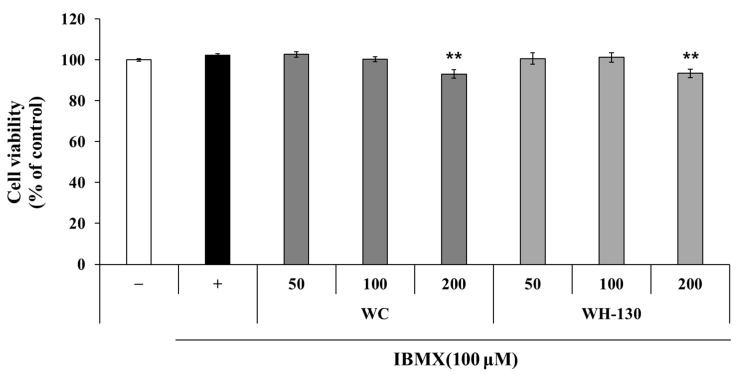
Cell viability associated with licorice extracts (WC and WH-130) was determined using the MTT assay. Data are expressed as mean ± SD (*n* = 3). ** *p* < 0.01.

As shown in Figure 3, licorice extracts did not affect cell viability at 50 or 100 μg/mL relative to the IBMX-treated control. In the 50 and 100 μg/mL treatments of all extracts, cell viability remained greater than 100%. Therefore, further experiments were performed using licorice extract concentrations of up to 100 μg/mL.

### 3.6. Effects of Licorice Extracts on Melanin Production in B16F10 Melanoma Cells

**Figure 4:**
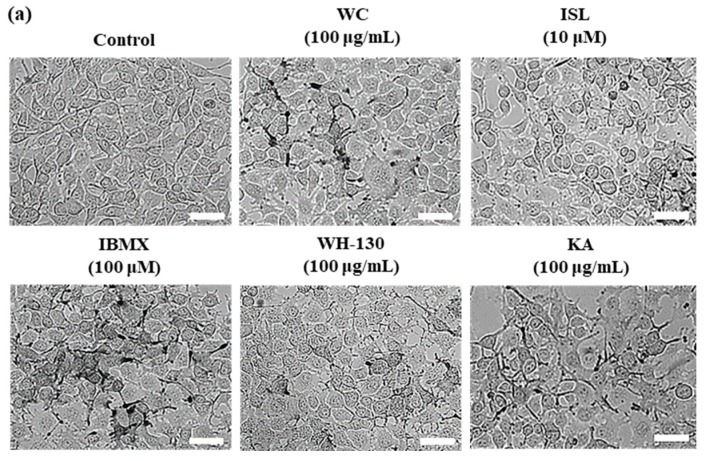

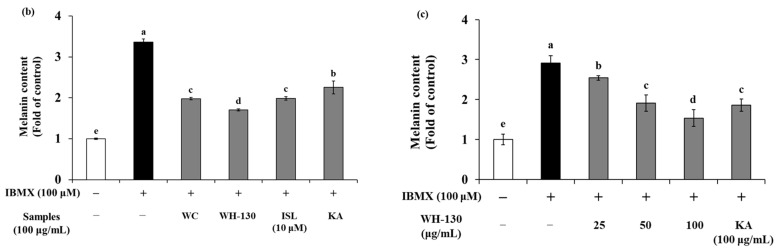
(**a**) Morphology of B16F10 melanoma cells treated with licorice extracts (WC and WH-130). Scale bar = 10 μm. (**b**) Effect of licorice extracts on IBMX-induced melanin synthesis in B16F10 melanoma cells. The values in both figures represent fold changes, compared to untreated control group. Data are expressed as mean ± SD (*n* = 3). Lowercase letters represent statistical differences at *p* < 0.05. WC, non-heated licorice; WC-130, heat-treated licorice (130 °C); ISL, isoliquiritigenin. (**c**) Inhibitory effect of WH-130 extracts at various concentrations (25, 50 and 100 μg/mL) on melanogenesis in B16F10 cells. KA (kojic acid, 100 μg/mL) was used as a positive control.

The pigmentation and melanin content of B16F10 cells are shown in Figure 4a,b. Melanin contents of B16F10 cells increased up to 3.4-fold with IBMX treatment relative to the untreated control. WC and WH-130 showed significantly decreased pigmentation and melanin content compared to the IBMX-treated group (Figure 4a,b). Both WC and WH-130 exhibited lower melanin content than KA-treated cells. The inhibitory effect of melanin synthesis in B16F10 cells treated with WH-130 was greater than WC. The amounts of melanin present after treatment with 100 μg/mL WC and WH-130 were reduced to approximately 0.59-fold and 0.51-fold, respectively, of the level in the IBMX-treated group. ISL (10 μM) and KA (100 μg/mL) suppressed melanin production to 0.59-fold and 0.67-fold, respectively of the level in IBMX-treated cells. 

This result shows that heat-treated licorice extract (WH-130) can inhibit melanin production more efficiently than non-heated extract (WC). Moreover, we observed that treatment with WH-130 extract at 25, 50 and 100 μg/mL (Figure 4c) resulted in a concentration-dependent decrease in the melanin content of B16F10 melanoma cells. Together, these results show that heating improved the anti-melanogenic activity of licorice extract.

### 3.7. Effects of Licorice Extracts on Tyrosinase Activity

#### 3.7.1. Mushroom Tyrosinase Activity

**Figure 5:**
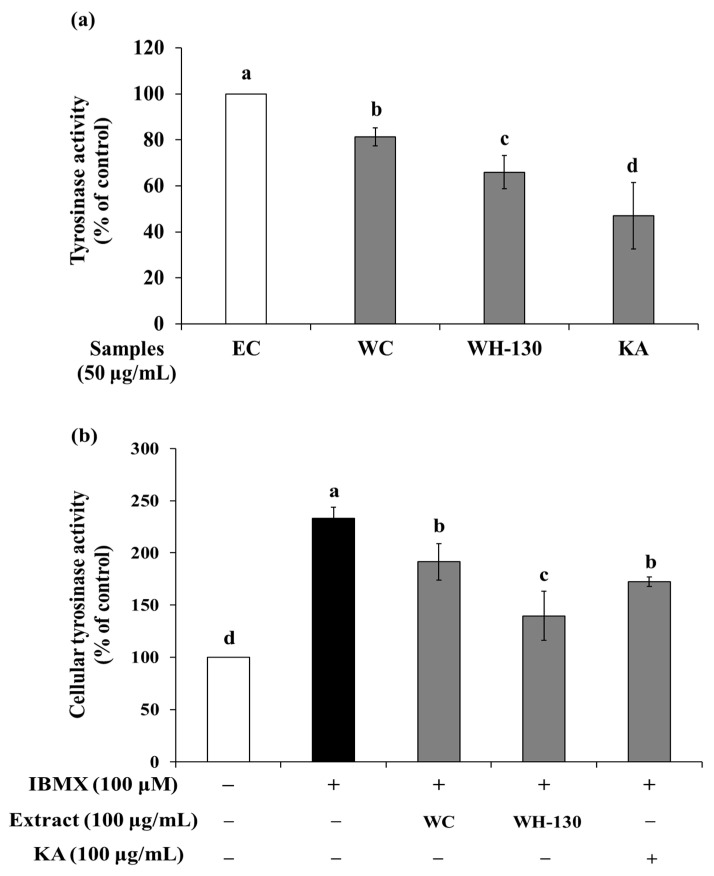
(**a**) Effects of licorice extracts (WC and WH-130) on mushroom tyrosinase activity. EC, enzyme control; KA, kojic acid. KA was used as a positive control. (**b**) Effects of licorice extracts (WC and WH-130) on cellular tyrosinase activity in B16F10 melanoma cells. Data are expressed as mean ± SD (*n* = 3). Lowercase letters represent statistical differences at *p* < 0.05.

The effect of licorice extracts on mushroom TYR activity is shown in Figure 5a. We confirmed that the effect of the extracts (WC and WH-130) on the oxidation of substrates by mushroom TYR occurred in cell-free conditions. At 50 μg/mL, WC extract inhibited enzyme activity to 81.33%, while WH-130 suppressed TYR activity to 65.95% of the EC level. Meanwhile, kojic acid (KA), a TYR inhibitor, caused inhibition of enzyme activity to 47.09% of the EC level. The inhibition of TYR activity was greater in heat-treated licorice (WH-130) than in non-heated licorice (WC).

#### 3.7.2. Cellular Tyrosinase Activity

As shown in Figure 5b, the TYR activity of IBMX-treated cells increased to 233% relative to the untreated control (100%). WC decreased TYR activity to 82.17% compared with the IBMX-treated group. WH-130 extract inhibited cellular TYR activity to 59.88% compared with IBMX-treated cells. KA reduced cellular TYR activity to 74.07% of the level in IBMX-treated cells. Together, these results show that licorice extracts (WC and WH-130) suppress TYR activity and that WH-130 has more potent TYR inhibitory activity than WC and KA.

Next, we investigated the correlations among TPC, ISL content, antioxidant activity and anti-melanogenic activity (Table 3). TPC was positively correlated with ISL content (0.827), as well as with ABTS^+^ and DPPH radical-scavenging activities (0.886 and 0.896, respectively), whereas TPC was inversely correlated with melanin content (–0.859). The ISL content was positively correlated with ABTS^+^ and DPPH radical-scavenging activities (0.977 and 0.985, respectively), whereas ISL content was inversely correlated with TYR activity (–0.834) and melanin content (–0.995). Antioxidant activities (ABTS^+^ and DPPH) were inversely correlated with TYR activity (–0.879 and –0.856, respectively) and melanin content (–0.974 and –0.984, respectively). These results indicate that phenolic compounds such as ISL in licorice are closely associated with the antioxidant and anti-melanogenic activities of licorice extracts.

### 3.8. Effects of Licorice Extracts on mRNA Expression of Melanogenesis-Related Genes

**Figure 6:**
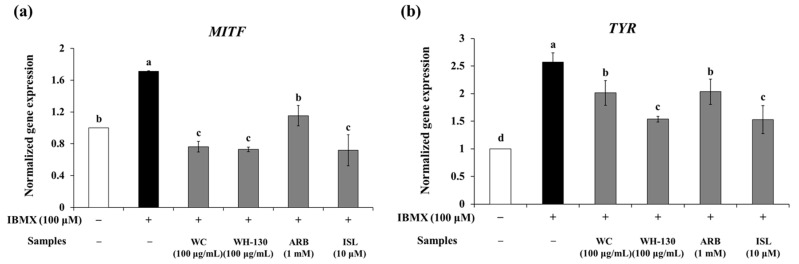

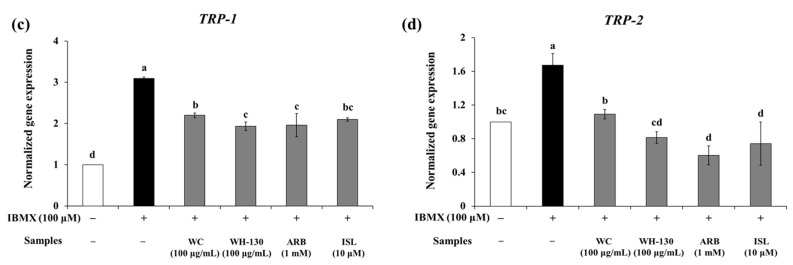
Effects of licorice extracts (WC and WH-130) on the expression of melanogenesis-related genes in B16F10 cells. (**a**) *MITF*, (**b**) *TYR*, (**c**) *TRP-1*, (**d**) *TRP-2*. Cells were treated with licorice extracts (WC and WH-130), arbutin (ARB, 1 mM) and isoliquiritigenin (ISL, 10 μM) for 48 h and the expression levels of genes including *MITF*, *Tyrosinase* (*TYR*), *TRP-1* and *TRP-2* were determined through a real-time PCR assay. ARB was used as a positive control. *β-actin* was used as a housekeeping gene control. Values are presented as mean ± SD from three experimental replicates. Lowercase letters represent statistical differences at *p* < 0.05.

We examined the expression of melanogenesis-related genes in B16F10 cells to elucidate the effects of licorice extracts on melanogenesis. Both WC and WH-130 suppressed the mRNA expression of *MITF*, *TYR*, *TRP-1* and *TRP-2* (Figure 6). WC inhibited the mRNA expression of *MITF*, *TYR*, *TRP-1* and *TRP-2* to levels 0.45-fold, 0.78-fold, 0.71-fold and 0.65-fold, respectively, of those in IBMX-treated cells. WH-130 inhibited the mRNA expression of *MITF*, *TYR*, *TRP-1* and *TRP-2* to 0.43-fold, 0.60-fold, 0.62-fold and 0.49-fold, respectively, of the IBMX-treated group levels. WH-130 extract had a stronger inhibitory effect on mRNA expression than WC extract. ISL also suppressed the expression of *MITF*, *TYR*, *TRP-1* and *TRP-2* (0.42-fold, 0.59-fold, 0.68-fold and 0.44-fold, respectively, relative to IBMX-treated cells). In the WH-130-treated group, the extent of the decrease in mRNA expression of melanogenesis-related genes was greater than in the WC-treated group, indicating that licorice extracts inhibit melanogenesis via suppression of the transcription of melanogenesis-related genes and that heat treatment improves the anti-melanogenic activity of licorice extract.

### 3.9. Effects of Licorice Extracts on Protein Expression of Melanogenesis-Related Genes

**Figure 7:**
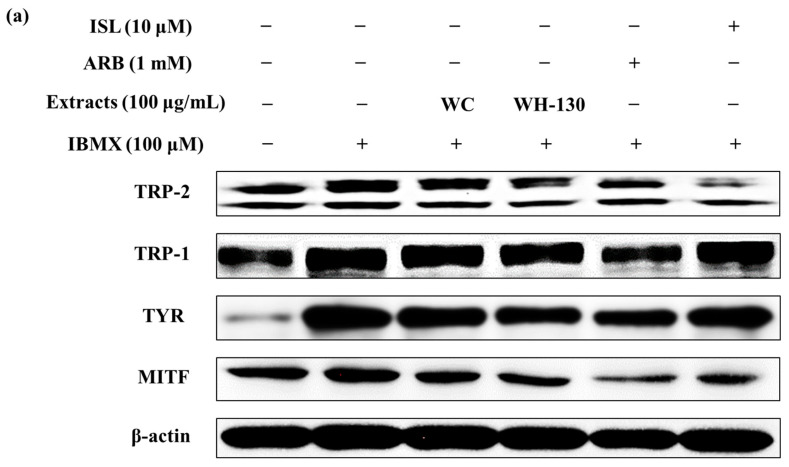

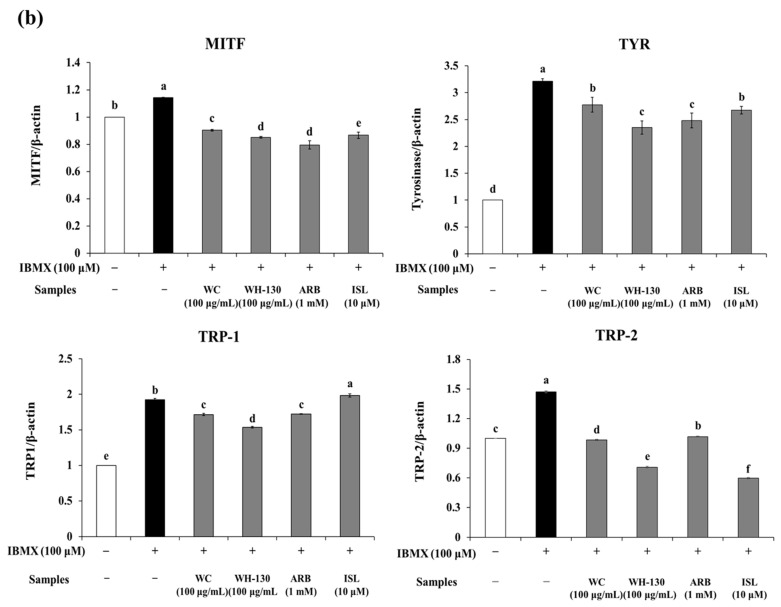
Effects of licorice extracts (WC and WH-130) on the expression of melanogenesis-related proteins in B16F10 cells. Cells were treated with licorice extracts (WC and WH-130), arbutin (ARB, 1 mM) and isoliquiritigenin (ISL, 10 μM) for 48 h. (**a**) Expression levels of proteins including MITF, TYR, TRP-1 and TRP-2 were determined through a western blotting assay (densitometric data). Equal protein loading was confirmed with β-actin as a housekeeping protein. (**b**) Values are presented as mean ± SD from three experimental replicates. Significance was statistically defined with small letters at *p* < 0.05.

As shown in Figure 7b, treatment with WC and WH-130 significantly reduced the protein expression levels of MITF, TYR, TRP-1 and TRP-2. Expression levels of MITF, TYR, TRP-1 and TRP-2 were suppressed in cells treated with WC (0.79-fold, 0.86-fold, 0.89-fold and 0.67-fold, respectively, relative to IBMX-treated cells). WH-130 showed reduced protein expression levels of MITF, TYR, TRP-1 and TRP-2 (0.74-fold, 0.73-fold, 0.80-fold and 0.48-fold, respectively, relative to IBMX-treated cells). ISL decreased the protein expression levels of MITF, TYR and TRP-2 (0.76-fold, 0.83-fold and 0.41-fold, respectively, relative to IBMX-treated cells). WH-130 suppressed the protein expression of melanogenesis-related genes more strongly than WC, showing that licorice extracts inhibit melanogenesis via suppression of the translation of melanogenesis-related genes and that heat treatment improves the anti-melanogenic activity of licorice extract.

## 4. Discussion

The results of this study demonstrate that heat treatment of licorice affects TPC, antioxidant activity and anti-melanogenic activity. WH-130 had higher browning index and TPC values than WC. Processing conditions, including temperature and pressure, affect the constituents of samples. [29]. Heating can promote Maillard reactions, which are linked to melanoidin production [23]. Melanoidins have some biological effects, including antioxidant, antitumor and antihypertensive activities, as well as blood sugar modulation [30]. These compounds are polymeric brown-colored macromolecules formed through the Maillard reaction from interactions between sugars and amino acids at high temperatures [21]. Thus, the more melanoidins in heated extract, the higher browning index. The color of non-heated licorice extract (WC) showed light brown, but the color was gradually darker with high temperature (130 °C). Heating process can also induce some changes in chemical structures of phenolic compounds and cause break down cell walls of plants, releasing phenolics, led to antioxidant activities [31]. Thus, the phenolic content of licorice extracts can increase with heat treatment [21]. Previous reports showed that heat treatment of honey increased melanoidin formation, and the degree of browning coincided with the increase in antioxidant activity [24]. Therefore, WH-130 showed higher antioxidant capacity than WC in the ABTS^+^ and DPPH radical-scavenging assays, which have been widely used to determine antiradical activities of samples based on the capacity to transfer electrons or hydrogen atoms [32].

The most common natural antioxidant and anti-melanogenic compounds are phenolics. Tricin and 9-hydroxyoctadecadienoic acid were identified as main phenolic compounds in *Sorghum bicolor*. In addition, these components showed antioxidant and anti-melanogenic effects by inhibition of tyrosinase activity [19]. In addition, *p*-coumaric acid, an active compound of *Sasa quelpaertensis* Nakai, has been proved that this compound strongly inhibited tyrosinase activity and reduced melanin production in melanoma cells [27]. According to previous research, ISL, a well-known flavonoid compound in the hydrolysis product of licorice root [6,7,8], exhibits antioxidant and anti-melanogenic activities by inhibition of TYR activity, melanosome transport and induction of melanin degradation [10,31]. So, we focused on ISL content of the extracts and ISL’s action in melanogenesis-related mechanisms. ISL is applied as a therapeutic agent for many diseases due to its numerous biological activities, including anti-inflammatory, anti-microbial, antioxidative, anti-cancer, immunoregulatory, hepatoprotective and cardioprotective effects [33]. In this study, a higher ISL content was measured in WH-130 than in WC. Skin hyperpigmentation is related to oxidative stresses. Free radical scavengers have been reported to play important roles in suppressing hyperpigmentation [17,34]. Together, these results show that heat treatment of licorice can improve its radical-scavenging activities and anti-melanogenic activities via increasing levels of antioxidant phenolic compounds such as ISL. According to previous literatures, there are glabrene, glabridin, glabrol, liquiritigenin in licorice (*Glycyrrhiza uralensis*, *G. glabra*), exhibiting tyrosinase inhibition. So, these compounds also may have an impact on anti-melanogenic activities [10,11,15,35]. Further studies on them are important to elucidate anti-melanogenic effects of licorice.

**Figure 8:**
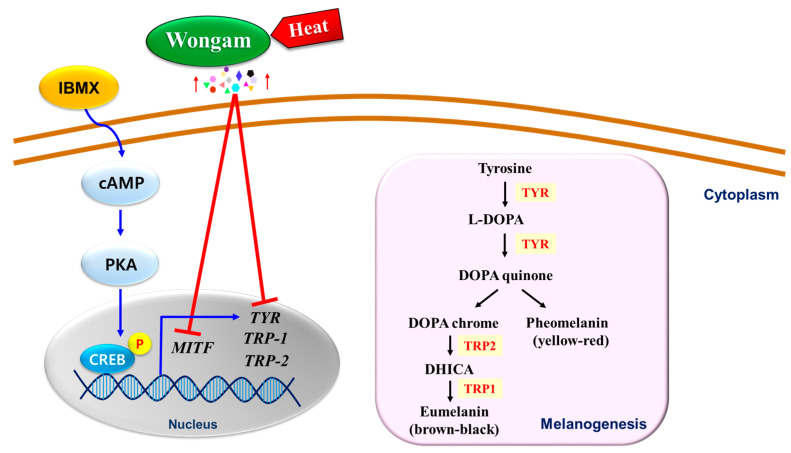
Schematic model of anti-melanogenic effects of heated Wongam in B16F10 melanoma cells. The red line and blue arrows indicate inhibition and stimulation, respectively. Heat treatment can increase the content of phenolic compounds such as ISL, linked to anti-melanogenic activities via regulating melanogenesis-related genes and proteins, in licorice.

Melanin synthesis is regulated by TYR, TRP-1, TRP-2 (dopachrome tautomerase) and MITF (Figure 8). TYR, a copper-containing glycoprotein, acts as the rate-limiting enzyme and plays a crucial role in melanin production. TYR catalyzes the hydroxylation of tyrosine to 3,4-dihydroxyphenylalanine (DOPA). TYR also catalyzes the oxidation of DOPA to dopaquinone and the conversion of dopaquinone to dopachrome. Then, dopachrome is converted to dihydro-indolizine (DHI) or indole 5,6-quinone-2-carboxylic acid (DHICA) [36,37,38]. In this pathway, TRP-2 catalyzes the conversion of dopachrome to DHICA, which is oxidized by TRP-1 to form eumelanin. MITF is a specific transcription factor that plays a critical role in melanogenesis as the major regulator of *TYR*, *TRP-1* and *TRP-2* expression (Figure 8) [28,37]. The chemical inducer of melanogenesis is 3-isobutyl-1-methylxanthine (IBMX), which increases TYR activity through the cAMP signaling pathway [13]. IBMX increases the intracellular cAMP concentration by inhibiting cAMP phosphodiesterase. The increase in cAMP levels activates protein kinase A (PKA) and activated PKA improves the activity and expression of cAMP-related binding protein (CREB) [39]. CREB binds to the *MITF* promoter, leading to upregulation of *MITF* gene expression. Activation of *MITF* promotes expression of TYR and TRPs [13,19]. Therefore, downregulation of *MITF* results in hypopigmentation. 

Heated licorice extract (WH-130) inhibited mushroom TYR and cellular TYR activity more effectively than non-heated licorice extract (WC). The inhibition of cellular TYR activity in licorice extracts may be caused by downregulation of *TYR*. These results are linked to the melanin content of B16F10 melanoma cells. In B16F10 cells, WH-130 treatment suppressed IBMX-induced melanin production better than WC treatment. Moreover, WH-130 downregulated the transcription and translation of melanogenesis-related markers (MITF, TYR, TRP-1, TRP-2) in B16F10 cells to a greater extent than WC. These markers may collectively contribute to overall anti-melanogenic activity [40].

Heating of licorice extract increased the abundance of antioxidant phenolic compounds such as ISL, and this increase was linked to increased antioxidant activities (ABTS^+^ and DPPH). Heated licorice extract (WH-130) also exhibited increased inhibitory activity of TYR and decreased melanin synthesis. There is a new trend in development of natural materials due to potential of safety. Licorice has been used in cosmeceutical creams for hyperpigmentation [41]. We demonstrated the potential of Wongam as a new skin-whitening agent for use in cosmetics and verified that heat treatment can improve its potential.

*Glycyrrhiza uralensis* Fischer (licorice) has antioxidant and UV photoprotective activities in human dermal fibroblasts [22]. However, the studies of comparison between Wongam (licorice) and other native species need to be conducted afterward. In addition, further studies on the unidentified components, such as glabrene, glabridin, of Wongam associated with its anti-melanogenic effect should be conducted using suitable methods. It is necessary to investigate the synergistic or antagonistic effects among phenolic compounds after analysis of constituents in licorice extract [40]. To examine how these substances acts on antioxidant and anti-melanogenic mechanisms, hydrogen atom transfer or single electron transfer systems and mitogen-activated protein kinase signaling pathway should be evaluated [32,37].

## 5. Conclusions

In this study, we investigated the potential of extracts of Wongam, a new cultivar of licorice, as a whitening agent for application in cosmetics and the effect of heat treatment on their bioactivity. Wongam extracts (WC and WH-130) showed high radical-scavenging activities and inhibited melanin synthesis in IBMX-induced melanocytes by suppressing the activity of TYR. Heat treatment increased the antioxidant and anti-melanogenic activities of Wongam. As a result, WH-130 showed superior antioxidative and anti-melanogenic activities than WC. Our results suggest that heat-treated Wongam extract (WH-130) is a potent pigment-reducing agent with possible applications in various dermatologic hyperpigmentation disorders, including brown spots, freckles and melasma, and show that thermal processing can be used to improve the anti-melanogenic activity of licorice.

## Figures and Tables

**Table 1:** List of primers used for the RT-PCR assay.

Gene Name		Sequence
*MITF* ^1^	Forward	GGCCAAGGCAGAGCAACTT
	Reverse	GCCCATGGTGGCAAGCT
*Tyrosinase*	Forward	ATAGGTGCATTGGCTTCTGG
	Reverse	CCAACGATCCCATTTTTCTT
*TRP* *-1* ^2^	Forward	GAGTGACATCCTGTGGCTCA
	Reverse	CGATACCCTGGGAACACTTT
*TRP-2*	Forward	GCTCCAAGTGGCTGTAGACC
	Reverse	AATGCAGTGGCTTGGAAATC
*β* *-actin*	Forward	CCCACTCCTAAGAGGAGGATG
	Reverse	AGGGAGACCAAAGCCTTCAT

^1^ MITF, microphthalmia-associated transcription factor; ^2^ TRP, tyrosinase-related protein.

**Table 2:** Content of isoliquiritigenin in licorice extracts.

Sample	Isoliquiritigenin (mg/g Extract, d.b.)
WC	1.354 ± 0.144 ^a^
WH-130	2.492 ± 0.152 ^b^

Lowercase letters (^a,b^) represent statistical differences at *p* < 0.05.

**Table 3:** Correlation analysis among antioxidant activities, TPC, isoliquiritigenin content and anti-melanogenic activity of licorice extracts.

Factors	TPC	ISL	ABTS^+^	DPPH	TYR	Melanin
TPC ^1^	1.000	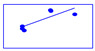	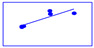	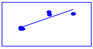	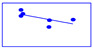	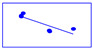
ISL ^2^	0.827 *	1.000	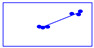	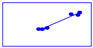	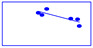	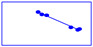
ABTS^+ 3^	0.886 *	0.977 ***	1.000	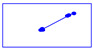	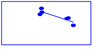	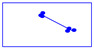
DPPH ^3^	0.896 *	0.985 ***	0.998 ***	1.000	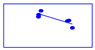	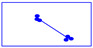
TYR ^4^	−0.662	−0.834 *	−0.879 *	−0.856 *	1.000	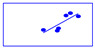
Melanin ^5^	−0.859 *	−0.995 ***	−0.974 **	−0.984 ***	0.821 *	1.000

^1^ TPC, total phenolic content; ^2^ ISL, isoliquiritigenin content of licorice extract, ^3^ ABTS^+^ and DPPH were calculated in mg of trolox equivalent per g. ^4^ TYR, tyrosinase activity in B16F10 melanoma cells. ^5^ Melanin, melanin content of B16F10 cells. Significance was determined using Pearson’s correlation coefficient; * *p* < 0.05, ** *p* < 0.01, *** *p* < 0.001.
